# Clinical, Hematologic, Biologic and Molecular Characteristics of Patients with Myeloproliferative Neoplasms and a Chronic Myelomonocytic Leukemia-Like Phenotype

**DOI:** 10.3390/cancers12071891

**Published:** 2020-07-14

**Authors:** Sonja Heibl, Bettina Gisslinger, Eva Jäger, Agnes Barna, Michael Gurbisz, Maike Stegemann, Peter Bettelheim, Sigrid Machherndl-Spandl, Michael Pfeilstöcker, Thomas Nösslinger, Gökhan Uyanik, Gregor Hoermann, Reinhard Stauder, Josef Thaler, Rajko Kusec, Peter Valent, Heinz Gisslinger, Klaus Geissler

**Affiliations:** 1 Department of Internal Medicine IV, Hospital Wels-Grieskirchen, 4600 Wels, Austria; sonja.heibl@klinikum-wegr.at (S.H.); josef.thaler@klinikum-wels.at (J.T.); 2 Division of Hematology and Hemostaseology, Department of Internal Medicine I, Medical University of Vienna, 1090 Vienna, Austria; bettina.gisslinger@meduniwien.ac.at (B.G.); peter.valent@meduniwien.ac.at (P.V.); heinz.gisslinger@meduniwien.ac.at (H.G.); 3 Department of Laboratory Medicine, Medical University of Vienna, 1090 Vienna, Austria; eva.jaeger@akhwien.at (E.J.); michael.gurbisz@meduniwien.ac.at (M.G.); gregor.hoermann@meduniwien.ac.at (G.H.); 4 Blood Transfusion Service, Blood Transfusion Service for Upper Austria, Austrian Red Cross, 4020 Linz, Austria; agnes.barna@o.roteskreuz.at; 5 Department of Internal Medicine V with Hematology, Oncology and Palliative Care, Hospital Hietzing, 1130 Vienna, Austria; maike.stegemann@wienkav.at; 6 Department of Internal Medicine I with Hematology with Stem Cell Transplantation, Hemostaseology and Medical Oncology, Ordensklinikum Linz Barmherzige Schwestern-Elisabethinen, 4020 Linz, Austria; peter@bettelheim.eu (P.B.); Sigrid.Machherndl-Spandl@elisabethinen.or.at (S.M.-S.); 7 Department of Internal Medicine III, Hanusch Hospital, 1140 Vienna, Austria; michael.pfeilstoecker@wgkk.at (M.P.); thomas.noesslinger@wgkk.at (T.N.); 8 Center for Medical Genetics, Hanusch Hospital, 1140 Vienna, Austria; goekhan.uyanik@wgkk.at; 9 Medical School, Sigmund Freud University, 1020 Vienna, Austria; 10 Central Institute of Medical and Chemical Laboratory Diagnostics, Medical University of Innsbruck, 6020 Innsbruck, Austria; 11 Internal Medicine V with Hematology and Oncology, Medical University of Innsbruck, 6020 Innsbruck, Austria; reinhard.stauder@i-med.ac.at; 12 School of Medicine, University of Zagreb, University Hospital Dubrava, 10000 Zagreb, Croatia; Rajko.Kusec@irb.hr; 13 Ludwig Boltzmann Institute for Hematology and Oncology (LBI HO), Medical University of Vienna, 1090 Vienna, Austria

**Keywords:** myeloproliferative neoplasm, chronic myelomonocytic leukemia, NGS, progenitor cells, prognosis

## Abstract

Patients with a myeloproliferative neoplasm (MPN) sometimes show a chronic myelomonocytic leukemia (CMML)-like phenotype but, according to the 2016 WHO classification, a documented history of an MPN excludes the diagnosis of CMML. Forty-one patients with an MPN (35 polycythemia vera (PV), 5 primary myelofibrosis, 1 essential thrombocythemia) and a CMML-like phenotype (MPN/CMML) were comprehensively characterized regarding clinical, hematologic, biologic and molecular features. The white blood cell counts in MPN/CMML patients were not different from CMML patients and PV patients. The hemoglobin values and platelet counts of these patients were higher than in CMML but lower than in PV, respectively. MPN/CMML patients showed myelomonocytic skewing, a typical in vitro feature of CMML but not of PV. The mutational landscape of MPN/CMML was not different from *JAK2*-mutated CMML. In two MPN/CMML patients, development of a CMML-like phenotype was associated with a decrease in the *JAK2* V617F allelic burden. Finally, the prognosis of MPN/CMML (median overall survival (OS) 27 months) was more similar to CMML (*JAK2*-mutated, 28 months; *JAK2*-nonmutated 29 months) than to PV (186 months). In conclusion, we show that patients with MPN and a CMML-like phenotype share more characteristics with CMML than with PV, which may be relevant for their classification and clinical management.

## 1. Introduction

Among the phenotypic diversity of patients with chronic myelomonocytic leukemia (CMML), peripheral blood (PB) monocytosis is a common prerequisite diagnostic criterion. In the 2016 WHO classification, persistent PB monocytosis ≥ 1 × 10^9^/L with monocytes accounting for ≥10% of the leukocytes is required to meet the criteria of CMML. Monocytosis, however, may also be found in a subset of patients with myeloproliferative neoplasms (MPN) detectable at the time of primary diagnosis or during the course of the disease. In patients with polycythemia vera (PV), monocytosis has been shown to be associated with a more unfavorable outcome [1]. Monocytosis is also a powerful and independent predictor of inferior survival in primary myelofibrosis (PMF) [2,3]. These findings indicate that there is a significant overlap between CMML and MPN, which is supported by the fact that CMML has been placed into the MPN/MDS category since 2000 [4].

In the 2016 WHO classification, patients with monocytosis and a history of MPN are explicitly excluded from the diagnosis of CMML and are categorized as MPN, even when they present with the typical phenotype of CMML [5]. The biological characteristics of this subgroup, however, are poorly investigated and it is unclear to which of both categories, MPN or CMML, these patients are more closely related. From a clinical point of view, better characterization of this subgroup would be important for more individualized patient management.

In this analysis, we describe the clinical, hematologic, molecular and biologic characteristics of a cohort of 41 patients with MPN and a CMML-like phenotype (monocytosis ≥ 1 G/L + monocytes ≥ 10% in PB) and compare it to the characteristics of patients with *JAK2*-mutated CMML and PV patients without a CMML-like phenotype, respectively.

## 2. Results

The first step of our analysis was to critically review patients with MPN regarding the presence of a CMML-like phenotype (monocytosis ≥ 1 G/L + monocytes ≥ 10% in PB). Among 585 MPN patients, 41 patients were classified as MPN with a CMML-like phenotype (MPN/CMML; 35 PV, 5 PMF, 1 ET). The hematologic characteristics of this group are given in Table 1 which also includes the hematologic parameters of PV patients without a CMML phenotype and CMML patients as a comparison. The age of patients with MPN/CMML (detailed features shown in Table S1) was higher than that of MPN patients (*p* < 0.001), but was not different from patients with CMML (*p* = 0.801). There was a male predominance in MPN patients with a CMML phenotype, which was not different from either MPN (*p* = 0.386) or CMML patients (*p* = 0.650). Phenotypically, patients with MPN/CMML had white blood cell counts (WBC) that were not different from CMML patients (*p* = 0.823) and PV patients (*p* = 0.058). The Hb values (*p* = 0.003) and platelet counts (*p* < 0.001) of these patients were higher than in CMML but lower than in PV (both *p* < 0.001), respectively.

The myelomonocytic skewing, as indicated by an inversed ratio of granulocyte-macrophage committed progenitor cells (CFU-GM) over erythroid committed progenitor cells (BFU-E) growth in semisolid cultures, is a typical in vitro characteristic of patients with CMML [6], but is only rarely found in patients with PV (K. Geissler). Therefore, it was of interest to analyze this biological feature in patients with MPN and a CMML-like phenotype. As shown in Table 2, in all patients in whom in vitro cultures could be performed, the number of CFU-GM was higher than the number of BFU-E, indicating myelomonocytic skewing in vitro. Moreover, myelomonocytic skewing was found in 85% of patients with CMML, in contrast to 9% of patients with PV. 

**Figure 1:**
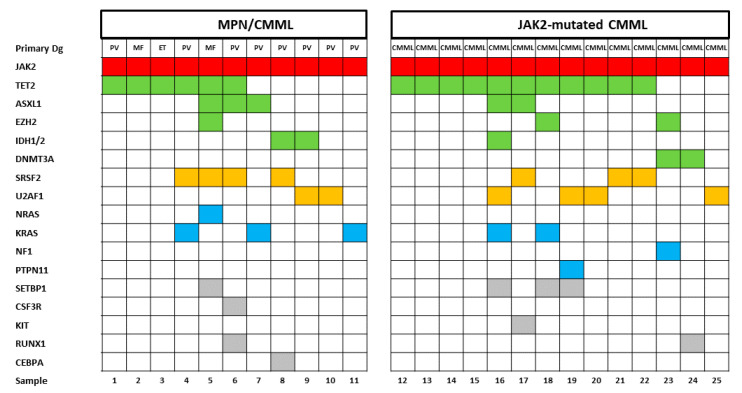
Mutational landscapes in patients with MPN/CMML and *JAK2*-mutated chronic myelomonocytic leukemia (CMML). Each column corresponds to one patient. Colored squares indicate mutated, white squares wild-type genes. The colors of mutant genes indicate the most affected functional categories. Red, green, yellow, blue, and grey represent the driver mutations, epigenetic regulators, spliceosome, RAS-pathway and other components, respectively. The variants of additional mutations are shown in Table S2.

The molecular aberrations in patients with CMML and PV have been published previously [7,8]. There may be some overlap, but the mutational landscapes of patients with CMML are usually different from those of patients with PV. Figure 1 shows the mutational landscape of patients with MPN and a CMML-like phenotype and in patients with *JAK2*-mutated CMML. Although the number of patients in each group is small, the category of mutated genes and their distribution, respectively, does not show any obvious difference in both groups.

**Figure 2:**
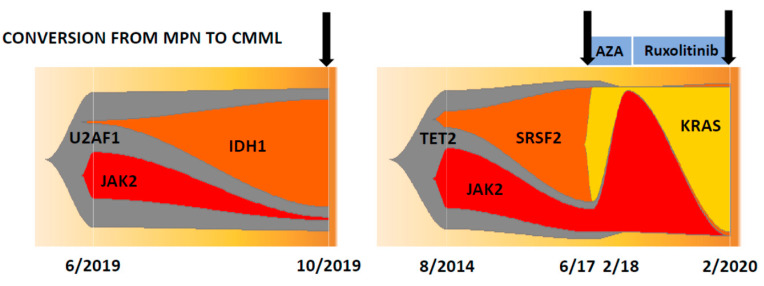
Clonal evolution in two patients with polycythemia vera who developed a CMML-like phenotype (indicated by arrows) during the course of their disease. The numbers indicate the month and year of NGS analysis.

In two patients in whom serial samples during the conversion from MPN to CMML were available, it was shown that the *JAK2* V617F variant allele frequency (VAF) in both patients markedly dropped, whereas the VAF in other genes that are commonly found in CMML concomitantly increased, suggesting that the CMML-like phenotype in these patients was driven by a genotype without the contribution of the *JAK2* mutation (Figure 2). In one of the patients, these genotypic changes that accompanied the development of CMML were observed twice. After the first conversion to CMML, the patient achieved remission after azacitidine treatment and regained his PV phenotype requiring phlebotomy. When the patient was treated with ruxolitinib, the *JAK2* V617F clone dropped again, but within several months of treatment the CMML-like phenotype returned, which was associated with increases in the VAF of *SRSF2* and *KRAS* mutations. Interestingly, in vitro colony formation from 10^5^ peripheral blood mononuclear cells (PBMNC) that was determined at these time points showed a CFU-GM/BFU-E ratio of 6/12 before conversion and ratios of 290/2 and 409/2, respectively, thereafter, indicating that the switch from MPN to CMML was associated with myelomonocytic skewing, which was shown by us in all other MPN patients with a CMML-like phenotype (Table 2).

**Figure 3:**
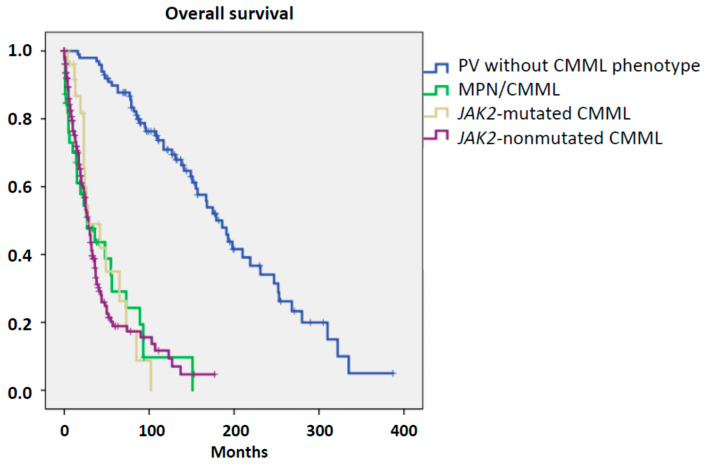
Overall survival in patients with myeloid disorder.

The prognosis of patients with hematological malignancies is an important aspect in the categorization of patients. Several reports indicated that the overall survival (OS) of patients with PV is much better than the OS of patients with CMML, although there is large heterogeneity depending on the presence or absence of risk factors [9,10]. We compared the OS of patients with MPN calculated either from diagnosis, if they showed monocytosis at diagnosis, or from the time when they developed a CMML-like phenotype and compared it with patients with *JAK2*-mutated CMML. Moreover, the survival of patients with PV without a CMML phenotype and patients with *JAK2*-nonmutated CMML is also given. The median overall survival of patients with MPN/CMML, *JAK2*-mutated CMML, *JAK2*-nonmutated CMML, and PV without a CMML phenotype was 27, 28, 29, and 186 months, respectively. Whereas there was no significant difference regarding survival between MPN/CMML, JAK2-mutated CMML, and JAK2-nonmutated CMML, respectively, all these cohorts had a significantly inferior survival as compared to PV without a CMML-like phenotype (Figure 3). Within the MPN/CMML cohort, patients who developed the CMML-like phenotype during the course of disease had a worse prognosis than patients with monocytosis at diagnosis (median OS 15 vs. 93 months; *p* < 0.001).

## 3. Discussion

Monocytosis is a hematologic feature that can be found in several reactive and clonal conditions. Persistent peripheral blood monocytosis ≥ 1 × 10^9^/L with monocytes accounting for ≥10% of the leukocytes is a hallmark of patients with CMML [5]. Such a CMML-like phenotype, however, can also be seen in a small subgroup of patients with MPN. Since this group of patients is poorly investigated, we characterized this patient group regarding hematologic, biologic, molecular and clinical features in order to define its place within the group of myeloid malignancies.

Normal hematopoietic function is maintained by a well-controlled balance of myelomonocytic, megaerythroid and lymphoid progenitor cell populations. This balance may be skewed in hematological malignancies, infections and autoimmunity [11,12,13,14,15,16,17]. Moreover, skewed hematopoiesis can be found in aged hematopoiesis [18]. Since semisolid in vitro cultures from the PBMNCs of normal individuals usually contain a higher concentration of erythroid colonies compared to myelomonocytic colony forming units, this test may be useful for investigating skewed differentiation towards the myelomonocytic over the erythroid commitment in patients. Myelomonocytic skewing in CMML was described by Itzykson and by us using the in vitro culture as a useful tool to study this phenomenon [6,19]. In this study, we show that all patients with MPN and a CMML-like phenotype showed myelomonocytic skewing and thus were biologically more related to CMML than to PV patients in whom this in vitro feature is only rarely observed.

In a limited number of patients, we had the opportunity to perform NGS in order to analyze the mutational landscape in MPN patients and monocytosis. It has been shown by several investigators that in CMML patients mutations involving *TET2* (~60%), *SRSF2* (~50%), *ASXL1* (~40%) and the oncogenic RAS pathway (~30%) are frequent [20]. In particular, the combination of *TET2* and *SRSF2* mutations is very frequently observed in CMML and highly specific for myeloid neoplasm with monocytosis [21,22]. On the other hand, *TET2/SRSF2* mutations were only found in 19%/1% of PV patients without monocytosis as compared to 57%/29% in PV patients with monocytosis ≥ 1 × 10^9^/L [1]. In our study, the mutational landscape in MPN patients with monocytosis was not clearly different from *JAK2*-mutated CMML patients and frequently showed mutation in genes of the epigenetic machinery, the spliceosome and the RAS pathway, which is more similar to CMML as compared to PV.

In two patients in whom serial samples were available, we were able to study the clonal evolution during the course of their disease. In both patients, the conversion from MPN to CMML was associated with a drop in the *JAK2* V617F clone and an increase in clones that are typically found in CMML, such as *SRSF2, KRAS* and *IDH1*. Of course, we cannot generalize this observation to all CMML-developing MPN patients. Indeed, there was a case report showing CMML as a transformation from PV and demonstrating that the CMML clone is most likely derived from the PV-JAK2 clone [23]. Moreover, in *JAK2*-mutated MPNs two routes of leukemic transformation have been demonstrated by analyzing 16 patients with a *JAK2*-mutant or *JAK2* wild-type acute myeloid leukemia after a *JAK2*-mutated MPN [24]. However, we can demonstrate at least in these two patients that the CMML-like phenotype was driven by a genotype without the contribution of the *JAK2* mutation.

The survival curves were not able to segregate MPN with a CMML-like phenotype from CMML, regardless if *JAK2*-mutated or nonmutated, but the survival of both groups was clearly different fromPV patients without monocytosis. The survival of the latter group was comparable to the survival of PV patients reported by other groups [8]. On the other hand, the median survival of *JAK2*-nonmutated CMML patients, which was also in the range of other published CMML cohorts, was not different from MPN patients with a CMML-like phenotype suggesting that with regard to prognosis this group was more similar to CMML than to PV.

Studies on MPN patients who develop monocytosis have been reported previously. In one study, the development of monocytosis was seen in 10 out of 237 patients with primary myelofibrosis (PMF) and indicated an accelerated phase of the disease [25]. Patients with “secondary CMML” had, as compared to PMF patients without monocytosis, increased WBC, and, in accordance with our findings, decreased hemoglobin, decreased platelet count, and more often circulating blasts. In another study, the *JAK2* V617F allelic burden was reported to be helpful in distinguishing CMML from PMF with monocytosis by showing a higher allelic burden in 11 cases of PMF with monocytosis as compared to seven CMML cases [26]. In our study, the allelic burden of MPN patients with a CMML-like phenotype was not different from patients with *JAK2*-mutant CMML. However, our study included more PV patients than PMF patients. In one study, flow cytometry-based subset analysis was useful to discriminate CMML from MPN with associated monocytosis [27]. Unfortunately, such data were not available in our patients to validate or not validate these findings. Finally, one study analyzed the clinical correlates, the prognostic impact and the survival outcome in 30 CMML patients with the *JAK2* V617F mutation [28]. As compared to 294 CMML patients without the *JAK2* V617F mutation, the authors reported some phenotypic changes but no difference in OS between the two groups, which can be confirmed in our study.

Understanding the pathogenesis and biology of the disease is important for the design of future treatment concepts. There are no systematically studied data on the effects of treatment in this patient group. What is known is that monocytosis is apparently an unfavorable outcome parameter in patients with PV and myelofibrosis (MF) as well [1,2,3]. Due to the unfavorable outcome, as well as the molecular and biologic similarities shared by patients with MPN with a CMML-like phenotype and *JAK2*-mutated CMML, it may be justified to consider our MPN/CMML patients as a special variant of CMML called CMML with concomitant myeloid neoplasm, as we have recently proposed [29]. This could be of clinical relevance if one speculates that treatment concepts that work in one group may also be promising in the other group. Unfortunately, effective treatment concepts have not been reported so far for either patient group. Due to the fact that these patients are rare, it might be, based on our data, justified to include both patient groups in studies with novel agents and/or novel combinations. In particular, the combination of a JAK2 inhibitor and hypomethylating agents may be an attractive concept for these patients.

## 4. Patients and Methods

The data from patients with MPN and a CMML-like phenotype were recruited from 4 centers that have a clinical focus on the management of hematologic malignancies including 2 centers from Vienna, one center from Wels and one center from Zagreb. The data from PV patients were used from an Austrian registry diagnosed for MPN according to the 2008 WHO diagnostic criteria between 1985 and 2020, which was created by clinicians and hematopathologists in the Departments of Hematology and Clinical Pathology at the Medical University of Vienna, Austria. The data from *JAK2*-mutated and nonmutated CMML patients were obtained from the Austrian Biodatabase for CMML (ABCMML), which has recently been shown to be a representative and useful real-life data source for further biomedical research [10]. The clinical and laboratory routine parameters were derived from patient records. Internal Review Board approval was obtained at each institution. Since this was a retrospective study including MPN patients from four different centers, a centralized review has not been performed. A detailed central manual retrospective chart review, however, was carried out to ensure data quality before analysis of data from the institutions. The data curation included the extraction of discrete data elements from patient records, a check for accuracy and consistency of data, and a verification that baseline data were reflective of MPN with a CMML phenotype, *JAK2*-mutated and nonmutated CMML, or PV without a CMML-like phenotype. Thus, data were obtained from 41 MPN patients with a CMML phenotype, 29 patients with *JAK2*-mutated CMML, 221 patients with *JAK2*-nonmutated CMML (total of 250 CMML patients), and 100 PV patients without a CMML-like phenotype. This research has been approved by the ethics committee of the City of Vienna on 10 June 2015 (ethic code: 15-059-VK).

### 4.1. Colony Assay

In one of our centers (the Medical University of Vienna), the assessment of hematopoietic colony formation in vitro has been an integral part of the diagnostic work up in patients with suspected myeloid malignancies for many years [30]. The number of circulating CFU-GM and BFU-E, respectively, was assessed in semisolid cultures, as previously described [31]. Mononuclear cells (MNCs) were isolated from the peripheral blood (PB) of patients by Ficoll–Hypaque density gradient centrifugation (density 1.077 g/mL, 400 *g* for 40 min). The low-density cells were collected from the interface between density solution and plasma, washed twice, and resuspended in Iscove’s modified Dulbecco’s medium (GIBCO, Paisley, Scotland). The PBMNCs were cultured in 0.9% methylcellulose, 30% fetal calf serum (FCS; INLIFE, Wiener Neudorf, Austria), 10% bovine serum albumin (Behring, Marburg, Germany), α-thioglycerol (10^−4^ mol/L) and Iscove’s modified Dulbecco‘s medium. For the stimulation of progenitor cells, cultures were supplemented with recombinant human granulocyte-macrophage colony-stimulating factor (GM-CSF) (10 ng/mL; R&D Systems, Minneapolis, MN, USA), rh-interleukin-3 (10 U/mL; Novartis, Basel, Switzerland) and erythropoietin (EPO, 2 U/mL; Roche, Basel, Switzerland). The cultures were plated in duplicates at 100 × 10^3^ PBMNC/mL. The plates were incubated at 37 °C, 5% CO_2_, and full humidity. After a culture period of 14 days, the cultures were examined under an inverted microscope. Aggregates with more than 40 translucent, dispersed cells were counted as CFU-GM. Bursts containing more than 100 red colored cells were scored as BFU-E. The progenitor cell data are expressed as mean values from the cultures.

### 4.2. Molecular Studies

The genomic DNA was isolated from the MNC fractions of these blood samples according to standard procedures. The mutational status of CMML-related protein coding genes was determined by targeted amplicon sequencing using the MiSeq platform (Illumina, San Diego, CA, USA). The details regarding gene panel, library preparation and data processing have been reported previously [10]. To minimize the chance to capture mutations that may be associated with non-neoplastic conditions, we chose a VAF cutoff of ≥20%, which has been shown to provide a specificity of 86% [22]. 

### 4.3. Statistical Analysis

The log-rank test was used to determine whether individual parameters were associated with the OS. The OS was defined as the time from sampling to death (uncensored) or last follow up (censored). The dichotomous variables were compared between different groups with the use of the chi-square test. The Mann–Whitney-U-test was used to compare 2 and the Kruskal–Wallis test was used to compare more than 2 unmatched groups when continuous variables were not normally distributed. The results were considered significant at *p* < 0.05. The statistical analyses were performed with the SPSS version 19.0.0 (SPSS Inc, Chicago, IL, USA) the reported *p* values were 2-sided. The evolution plots were generated according to Miller et al. [32].

## 5. Conclusions

In conclusion, we report here a comprehensive characterization of a cohort of MPN patients with a CMML-like phenotype. Our clinical, hematologic, biologic and molecular data demonstrate that these patients share many characteristics with CMML patients. This may be clinically relevant because our data justify considering this rare patient cohort as a special variant of CMML and including these patients in clinical trials that aim to improve the treatment options in CMML by using novel agents.

## Figures and Tables

**Table 1:** Hematologic Phenotype in Myeloid Disorders.

Parameter	MPN with CMML-Like Phenotype (*n* = 41)	CMML (*n* = 249)	PV without CMML-Like Phenotype (*n* = 99)
Age (years)	72 (48–93)	73 (36–93)	59 (25–82)
Sex (% male)	24/41 (59%)	155/249 (62%)	50/99 (51%)
White blood cell count (G/L)	13.4 (4.3–177)	13.1 (2.0–139)	11.1 (3.7–40)
Hemoglobin (g/dL)	13.4 (6.0–18.9)	11.1 (4.3–16.1)	17.1 (11.8–21.2)
Platelet count (G/L)	327 (13–1241)	108 (6–1181)	504 (151–2350)

MPN, myeloproliferative neoplasm; CMML, chronic myelomonocytic leukemia; PV, polycythemia vera.

**Table 2:** In Vitro Myelomonocytic Skewing in Myeloid Disorders.

Cohorts	Primary Diagnosis	CFU-GM/ 10^5^ MNC	BFU-E/ 10^5^ MNC	Ratio CFU-GM/ BFU-E; ≥1 (%)
Sample 1 (Pat. Nr. 1/2)	PV	120	105	1.14
Sample 2 (Pat. Nr. 1/9)	PV	21	11	1.91
Sample 3 (Pat. Nr. 1/10)	PV	290	2	145.00
Sample 4 (Pat. Nr. 1/12)	PV	16	7	2.29
Sample 5 (Pat. Nr. 1/14)	ET	431	190	2.47
Sample 6 (Pat. Nr. 1/16)	MF	255	119	2.14
Sample 7 (Pat. Nr. 4/9)	MF	720	148	4.86
Sample 8 (Pat. Nr. 4/10)	MF	67	32	2.1
Sample 9 (Pat. Nr. 4/11)	MF	288	12	24.00
Median (range) of total, *n* = 9	MPN/CMML	255 (1–720)	32 (2–190)	9/9 (100%)
Median (range) of total, *n* = 68	PV	18 (2–217)	65 (6–610)	6/68 (9%)
Median (range) of total, *n* = 88	CMML	44 (0–1958)	6 (0–300)	75/88 (85%)
Median (range) of total, *n* = 98	normal	9 (1–44)	33 (5–111)	1/98 (1%)

CFU-GM, colony forming-unit granulocyte/macrophage; BFU-E, burst forming-unit erythroid; MNC, mononuclear cells; PV, polycythemia vera; ET, essential thrombocythemia; MF, myelofibrosis; MPN, myeloproliferative neoplasm; CMML, chronic myelomonocytic leukemia.

## Supplementary Materials

The following are available online at https://www.mdpi.com/2072-6694/12/7/1891/s1, Table S1: Features of 41 patients with myeloproliferative neoplasms developing a chronic myelomonocytic leukemia-like phenotype, Table S2: Variants of additional mutations in patients with MPN/CMML and *JAK2*-mutated CMML.
