# Supplementary material for: Clinical, Hematologic, Biologic and Molecular Characteristics of Patients with Myeloproliferative Neoplasms and a Chronic Myelomonocytic Leukemia-Like Phenotype
Source: Cancers (Basel). 2020 Jul 14;12(7):1891. doi: 10.3390/cancers12071891 (PMC7409251; doi:10.3390/cancers12071891)
Supplement: Supplementary file 1 [file cancers-12-01891-s001.pdf]

Supplementary Materials: Clinical, Hematologic, Biologic and Molecular Characteristics of Patients with Myeloproliferative Neoplasms and a Chronic Myelomonocytic Leukemia-Like Phenotype

Sonja Heibl, Bettina Gisslinger, Eva Jäger, Agnes Barna, Michael Gurbisz, Maike Stegemann, Peter Bettelheim, Sigrid Machherndl-Spandl, Michael Pfeilstöcker, Thomas Nösslinger, Gökhan Uyanik, Gregor Hoermann, Reinhard Stauder, Josef Thaler, Rajko Kusec, Peter Valent, Heinz Gisslinger, Klaus Geissler

Table 1. Features of 41 patients with myeloproliferative neoplasms developing a chronic myelomonocytic leukemia-like phenotype.

Nr	Primary Diagn.	Age/sex	JAK2 Status	Cytoreductive Therapy	Date of Prim. Diagn.	Date of Conversion	Interval (Months)	Transf.	WBC G/L	Hb g/dl	HC %	PLT G/L	Mo %	AMC G/L
1/1	PV	63/m	NA	HU	4/07	3/09	23	no	15.5	15.4	45.4	668	10	1.6
1/2	PV	74/f	*JAK2 V617F*	-	8/18	3/20	19	no	9.4	13.3	46.9	388	13	1.2
1/3	PV	68/f	*JAK2 V617F*	-	12/16	12/16	0	no	21.5	14.3	44.6	1148	15	3.2
1/4	PV	80/m	*JAK2 V617F*	-	7/14	1/16	18	no	63.5	14.1	50.0	273	18	11.4
1/5	PV	51/m	*JAK2 V617F*	HU, ANA	5/14	3/16	22	no	16.8	10.8	38.5	575	14	2.4
1/6	PV	78/m	NA	-	2/13	12/18	70	no	18.9	8.8	24.6	275	10	1.9
1/7	PV	84/f	NA	HU	10/10	4/16	66	no	38.8	8.2	24.5	549	60	23.3
1/8	PV	72/f	*JAK2 V617F*	-	11/14	9/19	58	no	12.2	13.4	45.3	358	15	1.8
1/9	PV	73/m	*JAK2 V617F*	IFN	5/86	2/20	405	no	4.6	8.8	25.0	176	50	2.3
1/10	PV	67/m	*JAK2 V617F*	HU, AZA, RUX	8/14	12/19	64	no	29.6	10.3	31.1	225	12	3.6
1/11	PV	70/f	NA	-	4/04	4/04	0	no	33.5	12.3	44.5	13	20	6.7
1/12	PV	82/m	*JAK2 V617F*	Vyxeos	5/02	10/19	209	AML	4.4	8.6	25.4	72	54	2.4
1/13	PV	78/m	NA	HU, MEP, AZA	6/97	2/08	128	AML	13.4	16.7	50.8	116	10	1.3
1/14	ET	72/m	*JAK2 V617F*	IFN	12/99	3/11	135	no	28.7	13.5	41.6	352	14	4
1/15	PMF	77/m	neg	-	5/05	5/08	36	no	4.3	11.3	34.4	69	27	1.2
1/16	PMF	72/m	*JAK2 V617F*	HU, AZA, RUX	2/14	10/15	20	AML	176.8	6.6	22.3	86	10	17.7

2/1	PV	70/f	*JAK2 V617F*	HU	11/18	11/18	0	no	9.2	18.9	56.6	529	14	1,3
2/2	PV	61/m	*JAK2 V617F*	IFN, HU	6/18	6/18	0	no	23.3	17.4	51.9	286	13	3.0
2/3	PV	84/m	*JAK2 V617F*	HU, RUX	6/13	2/16	32	no	6.8	8.9	27.2	304	17	1.1
2/4	PV	86/f	*JAK2 V617F*	HU	4/17	4/17	0	no	7.1	16.2	54.7	333	17	1.2
2/5	PV	93/f	*JAK2 V617F*	HU	5/16	4/17	11	no	12.8	14.0	49.9	1241	12	1.5
2/6	PV	81/m	*JAK2 V617F*	HU	4/15	6/15	2	no	26.2	15.6	52.0	159	11	2.9
2/7	PV	70/m	*JAK2 V617F*	HU, AZA, LD-ARAC	4/11	6/13	26	AML	5.4	8.7	25.7	49	14	1.1
2/8	PV	70/m	NA	HU, AZA	1/10	5/16	76	AML	6.9	18.4	54.2	339	16	1.1
2/9	PV	84/f	*JAK2 V617F*	HU	5/09	2/15	69	no	10.1	14.8	44.1	465	12	1.3
2/10	PV	76/m	*JAK2 V617F*	HU	4/08	4/08	0	no	13.0	16.4	48.7	370	12	1.6
2/11	PV	79/f	neg	LEN, HU, RUX	3/07	7/09	28	no	16.9	9.7	31.0	205	18	3.0
2/12	PV	67/f	*JAK2 V617F*	HU, AZA	7/05	11/13	100	AML	5.2	6.0	17.0	83	30	1.6
2/13	PV	70/f	*JAK2 V617F*	IFN, HU, ANA, BUS, RUX	12/93	5/18	293	no	6.4	13.0	47.8	555	21	1.3
2/14	PV	69/f	*JAK2 V617F*	HU, RUX	7/82	3/18	428	no	30.2	14.1	46.7	92	15	3.9
3/1	PV	65/m	*JAK2 V617F*	HU	6/12	12/19	90	no	16.9	13.8	44.3	584	10	1.7
4/1	PV	56/m	*JAK2 V617F*	-	2/11	2/11	0	no	13.9	16.4	51.9	23	11	1.5
4/2	PV	70/f	*JAK2 V617F*	IFN	10/92	10/92	0	no	12.7	15.1	47.5	613	18	2.3
4/3	PV	57/m	*JAK2 V617F*	HU	10/10	10/10	0	no	19.2	16.3	47.4	667	11	2.1

4/5	PV	69/m	neg	-	5/10	1/15	56	no	9.2	11.2	34.3	321	12	1.1
4/6	PV	48/m	NA	-	4/97	11/00	43	no	14.0	14.7	43.9	699	11	1.5
4/7	PV	50/f	NA	TX	10/02	7/14	141	AML	12.7	14.4	38.0	327	11	1.4
4/8	PV	85/f	*JAK2 V617F*	HU	8/02	11/07	63	no	42.8	7.4	22.9	18	38	16.3
4/9	PMF	73/m	NA	-	2/91	5/98	87	AML	34.5	8.9	28.5	336	10	3.5
4/10	PMF	72/f	*JAK2 V617F*	IM, HU	5/99	11/00	18	no	12.8	13.0	38.8	490	32	4.1
4/11	PMF	67/m	NA	-	7/98	10/98	3	AML	10.5	9.1	28.5	52	25	2.6

CMML, chronic myelomonocytic leukemia; PV, polycythemia vera; ET, essential thrombocythemia; PMF, primary myelofibrosis; NA, not available; HU, hydoxyurea; ANA, anagrelide; IFN, interferon; AZA, azacitidine; RUX, ruxolitinib; MEP, meprobaman; LD-ARAC, low dose cytarabine; BUS, busulfan; TX, transplantation; IM, imatinib; AML, acute myeloid leukemia; WBC, white blood cell count; Hb, hemoglobin; HC, hematocrite; PLT, platelet value; Mo, monocytes; AMC, absolute monocyte count. The blood pictures in this table are at the time when patients developed a CMML-like phenotype.

Table S2. Variants of additional mutations in patients with MPN/CMML and *JAK2*-mutated CMML.

Sample	Gene	cDNA Change	Amino Acid Change	Variant Allele Frequency
**MPN/CMML**				
1	*JAK2*	c.1849G > T	p.Val617Phe	79.5%
	TET2	c.5707C > T	p.Gln1903*	46.9%
	TET2	c.2626C > T	p.Gln876*	46.0%
2	*JAK2*	c.1849G > T	p.Val617Phe	44.2%
	TET2	c. 5473C > T	p.Gln1825*	43.4%
	TET2	c.2746C > T	p.Gln916*	42.2%
3	*JAK2*	c.1849G > T	p.Val617Phe	68.5%
	TET2	c.1648C > T	p.Arg550*	36.4%
	TET2	c.3508C > T	p.Gln1170*	34.4%
4	*JAK2*	c.1849G > T	p.Val617Phe	7.3%
	TET2	c.2200dupC	p.Gln734Profs*20	44.5%
	TET2	c.5500C > T	p.Gln1834*	42.0%
	SRSF2	c.284C > A	p.Pro95His	44.7%
	KRAS	c.35G > T	p.Gly12Val	42.8%
5	*JAK2*	c.1849G > T	p.Val617Phe	78.1%
	TET2	c.1064G > A	p.Gly376Asp	62.2%
	ASXL1	c.2261C > A	p.Glu635fs	38.0%
	EZH2	c.553G > C	p.Asp185His	36.0%
	SRSF2	c.284C > T	p.Pro95Leu	49.0%
	NRAS	c.35G > A	p.Gly12Asp	28.5%
	SETBP1	c.691/G > C	p.Val231Leu	74.3%
6	*JAK2*	c.1849G > T	p.Val617Phe	46.0%
	TET2	c.3094C > G	p.Leu1032Val	50.2%
	ASXL1	c.3217C > T	p.Arg1073Cys	52.7%
	SRSF2	c.284C > A	p.Pro95His	46.3%
	CSF3R	c.1213G > A	p.Glu405Lys	51.6%
	RUNX1	c.898delA	p.Thr300Rfs*11	46.5%
7	*JAK2*	c.1849G > T	p.Val617Phe	96.7%
	ASXL1	c.2077C > T	p.Arg693*	49.5%
	KRAS	c.35G > A	p.Gly12Asp	28.1%
8	*JAK2*	c.1849G > T	p.Val617Phe	39.6%
	IDH2	c.419G > A	p.Arg140Gln	44.6%
	SRSF2	c.283C > A	p.Pro95Thr	45.0%
	CEBPA	c.584_589dupACCCGC	p.His195.Pro196dup	43.5%
9	*JAK2*	c.1849G > T	p.Val617Phe	<5.0%
	IDH1	c.394C > T	p.Arg132Cys	32.3%
	U2AF1	c.101C > T	p.Ser34Phe	42.1%
10	*JAK2*	c.1849G > T	p.Val617Phe	7.0%
	U2AF1	c.101C > A	p.Ser34Tyr	42.3%
11	*JAK2*	c.1849G > T	p.Val617Phe	33.6%
	KRAS	c.34G > C	p.Gly12Arg	24.5%
***JAK2*-mutated CMML**				
12	*JAK2*	c.1849G > T	p.Val617Phe	91.6%
	TET2	c.3541G > A	p.Val1181Ile	40.7%
13	*JAK2*	c.1849G > T	p.Val617Phe	8.9%
	TET2	c.4579C > T	p.Gln1527*	48.0%
	SRSF2	c.284C > T	p.Pro95Leu	48.7%
	KIT	c.2447A > T	p.Asp816Val	27.5%
14	*JAK2*	c.1849G > T	p.Val617Phe	43.6%
	TET2	c.4223A > G	p.Asn1387Ser	48.3%
	TET2	c.1608A > G	p.Ile1762Val	52.3%
15	*JAK2*	c.1849G > T	p.Val617Phe	31.2%

	TET2	c.5284A > G	p.Ile1762Val	52.1%
16	*JAK2*	c.1849G > T	p.Val617Phe	42.3%
	TET2	c.2599T > C	p.Tyr867His	48.7%
	ASXL1	c.2678G > A	p.Trp893*	41.5%
	IDH2	c.44G > A	p.Arg88Gln	38.0%
	U2AF1	c.248G > A	p.Arg83His	36.3%
	SETBP1	c.691G > C	p.Val231Leu	47.3%
17	*JAK2*	c.1849G > T	p.Val617Phe	26.2%
	TET2	c.5018delC	p.Pro1673Hisfs*22	45.2%
	ASXL1	c.2757dupA	p.Pro920Thrfs*4	43.2%
	SRSF2	c.284C > A	p.Pro95His	47.3%
18	*JAK2*	c.1849G > T	p.Val617Phe	29.3%
	TET2	c.191T > G	p.Cys1289Trp	49.5%
	EZH2	c.1720G > A	p.Gly574Arg	48.7%
	SETBP1	c.691/G > C	p.Val231Leu	49.0%
	KRAS	c.179G > T	p.Gly60Val	48.4%
19	*JAK2*	c.1849G > T	p.Val617Phe	48.8%
	TET2	c.5347A > G	p.Ile1783Val	98.0%
	U2AF1	c.251A > C	p.Gln157Pro	35.1%
	SETBP1	c.2602G > A	p.Asp868Asn	40.7%
	PTPN11	c.1530G > T	p.Gln510His	38.8%
20	*JAK2*	c.1849G > T	p.Val617Phe	34.9%
	TET2	c.1608A > G	p.Ile1762Val	50.0%
	U2AF1	c.251A > G	p.Gln84Arg	50.0%
21	*JAK2*	c.1849G > T	p.Val617Phe	89.9%
	TET2	c.1486T > G	p.Leu1721Trp	50.0%
	TET2	c.3632G > A	p.Cys1211Tyr	47.5%
	SRSF2	c.284C > A	p.Pro95His	43.7%
22	*JAK2*	c.1849G > T	p.Val617Phe	49.5%
	TET2	c.3430dupG	p.Glu1144Glyfs*	44.5%
	SRSF2	c.284C > A	p.Pro95His	47.1%
23	*JAK2*	c.1849G > T	p.Val617Phe	91.2%
	EZH2	c.553G > C	p.Asdp185His	66.6%
	DNMT3A	c.2077C > T	p.Arg693Cys	36.4%
	NF1	c.4367G > C	p.Arg1456Thr	79.3%
24	*JAK2*	c.1849G > T	p.Val617Phe	6.4%
	DNMT3A	c.2204A > G	p.Tyr735Cys	23.1%
	DNMT3A	c.2095G > T	p.Gly699Cys	22.6%
	RUNX1	c.167T > C	p.Leu56Ser	46.8%
25	*JAK2*	c.1849G > T	p.Val617Phe	85.3%
	U2AF1	c.101C > A	p.Ser34Tyr	38.0%

© 2020 by the authors. Licensee MDPI, Basel, Switzerland. This article is an open access article distributed under the terms and conditions of the Creative Commons Attribution (CC BY) license (http://creativecommons.org/licenses/by/4.0/).
